# CD19 CAR-T细胞治疗B淋巴母细胞淋巴瘤并发DIC 1例报告并文献复习

**DOI:** 10.3760/cma.j.cn121090-20241202-00523

**Published:** 2024-12

**Authors:** 莹莹 李, 沛儒 李, 慧雯 江, 恒 梅

**Affiliations:** 1 华中科技大学同济医学院附属协和医院，武汉 430022 Institute of Hematology, Union Hospital, Tongji Medical College, Huazhong University of Science and Technology, Wuhan 430022, China; 2 湖北省血栓与止血临床医学研究中心，武汉 430022 Hubei Clinical and Research Centre of Thrombosis and Hemostasis, Wuhan 430022, China

**Keywords:** CAR-T细胞治疗, B淋巴母细胞淋巴瘤, 弥散性血管内凝血, CAR-T therapy, B-lymphoblastic lymphoma, Disseminated intravascular coagulation

## Abstract

**目的:**

探讨嵌合抗原受体T细胞（CAR-T细胞）治疗B淋巴母细胞淋巴瘤后并发DIC的临床表现、发生机制和诊疗方案。

**方法:**

回顾性收集2020年4月于华中科技大学同济医学院附属协和医院血液科接受CAR-T细胞治疗的1例B淋巴母细胞淋巴瘤患者的临床资料并进行分析。以“嵌合抗原受体T细胞治疗”“凝血”“出血”“血栓”“CAR-T”“coagulation”“bleeding”“thrombosis”为检索词，检索建库至2024年11月中文数据库（中国知网数据库、万方数据库）及Pubmed数据库进行文献复习。

**结果:**

患者为32岁男性，确诊B淋巴母细胞淋巴瘤10个月余，在3个周期化疗及异基因造血干细胞移植后复发，骨髓细胞学提示20.24％为表型异常的B系原始细胞。回输CAR-T细胞4×10^6^/kg后第2天，患者出现2级细胞因子释放综合征（CRS），第4天发生鼻出血，第9天再发高热，凝血指标恶化，并发DIC、持续性低纤维蛋白原血症。予以托珠单抗、糖皮质激素拮抗CRS，输注血制品及补充纤维蛋白原积极替代治疗后，患者CRS及凝血异常逐渐恢复。CAR-T细胞治疗后规律随访16个月，体内CAR-T细胞持续存在，骨髓流式细胞术MRD维持阴性。后因患者自行停用口服抗排异药物，排异加重合并感染，患者及家属要求出院而失访。文献检索共检索到相关英文文献20篇，中文文献6篇。

**结论:**

CAR-T细胞治疗相关出凝血障碍伴随CRS发生，低纤维蛋白原血症是其特征，进展至DIC时危及生命，拮抗CRS和替代治疗是有效治疗手段。

## Abstract (en)

**Objective:**

To explore the clinical manifestations, pathogenesis, and therapeutic approaches of disseminated intravascular coagulation（DIC）associated with chimeric antigen receptor T-cell（CAR-T）therapy for B-lymphoblastic lymphoma.

**Methods:**

Retrospective collection and analysis were conducted on the clinical data of a patient with B-lymphoblastic lymphoma who received CAR-T therapy in the Hematology Department of Union Hospital, Tongji Medical College, Huazhong University of Science and Technology, in April 2020. To review the literatures, Chinese databases（CNKI, Wanfang Database）and PubMed were searched form database inception up to November 2024 using the retrieval entries “chimeric antigen receptor T-cell therapy”, “CAR-T”, “coagulation”, “bleeding”, and “thrombosis”.

**Results:**

The patient, a 32-year-old male, diagnosed with B-lymphoblastic lymphoma for over 10 months, relapsed after three cycles of chemotherapy and allogeneic hematopoietic stem cell transplantation, with bone marrow cytology indicating 20.24％ abnormal phenotype B-lineage precursor cells. After CAR-T cell infusion at a dose of 4×10^6^/kg, the patient developed grade 2 cytokine release syndrome（CRS）on day 2, nasal bleeding on day 4, high fever again on day 9, with worsening coagulation parameters, DIC and persistent hypofibrinogenemia. After treatment with tocilizumab, corticosteroids to counteract CRS, and active replacement therapy such as administration of blood product and fibrinogen, the patient's CRS and coagulation abnormalities gradually improved. The patient was followed up for 16 months regularly after CAR-T therapy, with CAR-T cells sustained and minimal residual disease remained negative as assessed by bone marrow flow cytometry. Subsequently, the patient stopped taking oral immunosuppressants on his own, which aggravated rejection reaction complicated with infection. The patient and his family requested to be discharged and lost the follow-up. A total of 20 relevant English articles and 6 Chinese articles were retrieved from the literature search.

**Conclusion:**

CAR-T-associated coagulopathy occurs alongside CRS. It is characterized by hypofibrinogenemia, and is life-threatening when progressing to DIC. Anti-CRS and replacement therapies have proven to be effective treatment strategies.

B淋巴母细胞淋巴瘤（B-lymphoblastic lymphoma，B-LBL）是一种起源于前体B淋巴细胞的高侵袭性非霍奇金淋巴瘤（non-Hodgkin's lymphoma, NHL），发生率占NHL的4.5％[1]。复发/难治性B-LBL病情进展迅速，总体生存率仅10％～30％[2]。近年来，嵌合抗原受体T细胞（chimeric antigen receptor T cell，CAR-T细胞）疗法在血液肿瘤的治疗中取得了显著进展，在复发/难治性淋巴瘤患者中的完全缓解率可以达到54％～100％[3]。需要注意的是，CAR-T细胞治疗也可能引起相关并发症，如细胞因子释放综合征（cytokine release syndrome，CRS）和出凝血功能障碍，后者严重时可发展为弥散性血管内凝血（DIC），威胁患者生命[4]–[5]。本文报道１例复发/难治性B-LBL患者接受CAR-T细胞治疗后出现典型DIC的病例，并进行文献复习。

## 病例资料

患者，男，32岁，因“确诊淋巴瘤10个月余，复发6 d”于2021年2月3日入院。患者入院前10个月余因右侧腮腺持续肿大于当地医院就诊，腮腺病理：CD99（+）、TdT（−）、CD20（+）、CD43（+）、CD79a（+）、CyclinD1（−）、SOX11（−）、Ki67（LI：50％），考虑为B-LBL；骨髓细胞学：原始淋巴细胞85.5％；骨髓流式免疫分型：83.8％为异常幼稚B淋巴细胞，表达CD10、CD19、TDT、CD22、HLA-DR，部分表达CD38、CD13、CD20、CD123、CD79a；荧光原位杂交：17p13缺失；PET-CT：双侧颈后、耳后、腮腺内及腮腺旁、颈部双侧、左上臂皮下、双侧锁骨上区、左侧肩部皮下、双侧腋窝区、双侧胸小肌旁、纵隔内、腹盆腔及双侧腹股沟区多发淋巴结；脾脏肿大、双肾肿大，代谢弥漫性增高，全身骨髓代谢弥漫性增高。当地医院诊断为B-LBL（Ⅳ期，TP53突变，高危组），给予2次化疗，具体方案不详。2020年10月1日于我院接受HR1方案化疗，达到完全缓解。2020年11月28日于我院行亲缘全相合异基因造血干细胞移植，1个月后复查骨穿，骨髓细胞学及骨髓流式细胞术微小残留病（MRD）均未见异常B系幼稚细胞，荧光原位杂交未见异常信号细胞，提示TP53转阴。2021年1月29日复查骨穿，骨髓细胞学：20.24％为表型异常的B系原始细胞；荧光原位杂交：17p13缺失；嵌合度检测：混合嵌合型，供者嵌合率57.5％。入院查体：体温37.8°C，脉率132次/min，呼吸20次/min，血压109/75 mmHg，美国东部肿瘤协作组评分为3分，慢性病容，巩膜轻度黄染，全身皮肤色素沉着伴脱屑，肺部呼吸音粗，两肺底闻及少许湿啰音，双下肢轻度凹陷性水肿。实验室检查：WBC 1.49×10^9^/L，HGB107 g/L，PLT 43×10^9^/L，中性粒细胞0.82×10^9^/L。初步诊断：①B-LBL（Ⅳ期，TP53突变，高危组）；②造血干细胞移植状态；③急性移植物抗宿主病。入院后给予泽布替尼控制原发病，甲泼尼龙抗排异反应，同时给予抗感染、退热等对症支持治疗。患者符合人源化抗CD19 CAR-T临床试验（NCT04008251）纳入标准，签署知情同意书后，采集原移植供者单个核细胞用于制备CAR-T细胞。患者于2021年3月7日开始预处理化疗（氟达拉滨30 mg/m^2^第1～3天，环磷酰胺750 mg/m^2^第3天），在预处理后再次出现粒细胞缺乏（粒缺）伴发热，经多轮抗感染方案调整后，3月26日体温基本恢复正常。回输前复查骨穿，细胞学提示原始细胞占88.01％。与家属沟通病情及回输CAR-T细胞相关注意事项，于4月2日至3日共回输CAR-T细胞4×10^6^/kg。回输第1天，患者发热至38.5 °C，可自行退热，WBC 0.83×10^9^/L，PLT 20×10^9^/L，中性粒细胞0.37×10^9^/L。因患者处于持续粒缺状态，继续维持头孢他啶阿维巴坦联合替加环素抗感染，卡泊芬净联合泊沙康唑预防真菌。回输后第1至2天患者持续高热，并出现一过性低血压，白细胞介素-6（IL-6）57.46 pg/ml。根据美国移植与细胞治疗学会分级标准[5]，诊断为2级CRS，给予托珠单抗8 mg/kg治疗后患者体温峰值降低。第4天患者出现鼻出血，PLT 15×10^9^/L，活化部分凝血活酶时间（APTT）57.4 s，纤维蛋白原1.5 g/L，给予血小板和血浆输注。第5天，D-二聚体1.08 mg/L FEU，APTT 55.4 s，纤维蛋白原1.15 g/L，给予纤维蛋白原输注。第7天复查骨穿评估疗效，骨髓流式MRD示58.01％为异常B系原始细胞，骨髓CAR-T细胞比例为4.9％。第9天患者再次高热，体温39.6 °C，IL-6为1925 pg/ml，而C反应蛋白处于正常范围，提示此次发热由CRS引起可能性大。给予托珠单抗及地塞米松治疗后，患者体温高峰下降，但是凝血功能持续恶化，APTT 62.9 s，凝血酶原时间（PT）19.1 s，纤维蛋白原0.87 g/L，D-二聚体15.86 mg/L FEU，纤维蛋白降解产物84.4 µg/ml。根据中国DIC诊断积分系统[6]，该患者第9至10天可诊断为DIC（表1），在继续抗感染及抗CRS的基础上，予以输注血制品及补充纤维蛋白原。经治疗后，患者凝血指标逐渐好转，其中纤维蛋白原恢复较为缓慢（图1）。CAR-T回输后第21天复查骨穿，流式细胞术MRD未见异常B系原始细胞；2个月复查骨穿，嵌合度达到100％；3个月复查骨穿，TP53基因转阴；规律随访至16个月，患者体内CAR-T细胞持续存在（图2），骨髓流式细胞术MRD持续阴性。后患者自行停用口服抗排异药物，因排异加重再次入院治疗，但患者及家属因重度排异反应且合并重症感染而要求出院，随后失访。

**表1:** 该患者在CAR-T细胞回输后的中国DIC诊断积分系统评分

CDSS积分系统	分数	时间（回输后天数）											
		4	5	6	7	8	9	10	11	12	13	14	28
1.基础疾病	2	2	2	2	2	2	2	2	2	2	2	2	2
2.临床表现（满足其一）	1	0	0	0	0	0	0	0	0	0	0	0	0
（1）与原发病无关的休克或微循环障碍													
（2）无法解释的器官衰竭													
3.实验室指标													
（1）PLT													
≥50×10^9^/L	0												
<50×10^9^/L	1	1	1	1	1	1	1	1	1	1	1	1	1
24 h内下降大于50％	1												
（2）D-二聚体													
<5 mg/L	0	0	0	0	0	0					0	0	0
5～9 mg/L	2						2		2	2			
≥9 mg/L	3							3					
（3）PT及APTT延长													
PT延长<3 s且APTT延长<10 s	0				0	0			0	0	0	0	0
PT延长≥3 s或APTT延长≥10 s	1	1	1	1			1	1					
PT延长≥6 s	2												
4.纤维蛋白原													
≥1.0 g/L	0	0	0	0	0	0			0	0			0
<1.0 g/L	1						1	1			1	1	
总分（≥6分即可诊断DIC）		4	4	4	3	3	7	8	5	5	4	4	3

**注** CDSS：中国弥散性血管内凝血诊断积分系统；PT：凝血酶原时间；APTT：活化部分凝血活酶时间；DIC：弥散性血管内凝血。根据CDSS积分系统，该患者在CAR-T细胞回输后第9至10天可诊断为DIC

**图1:**
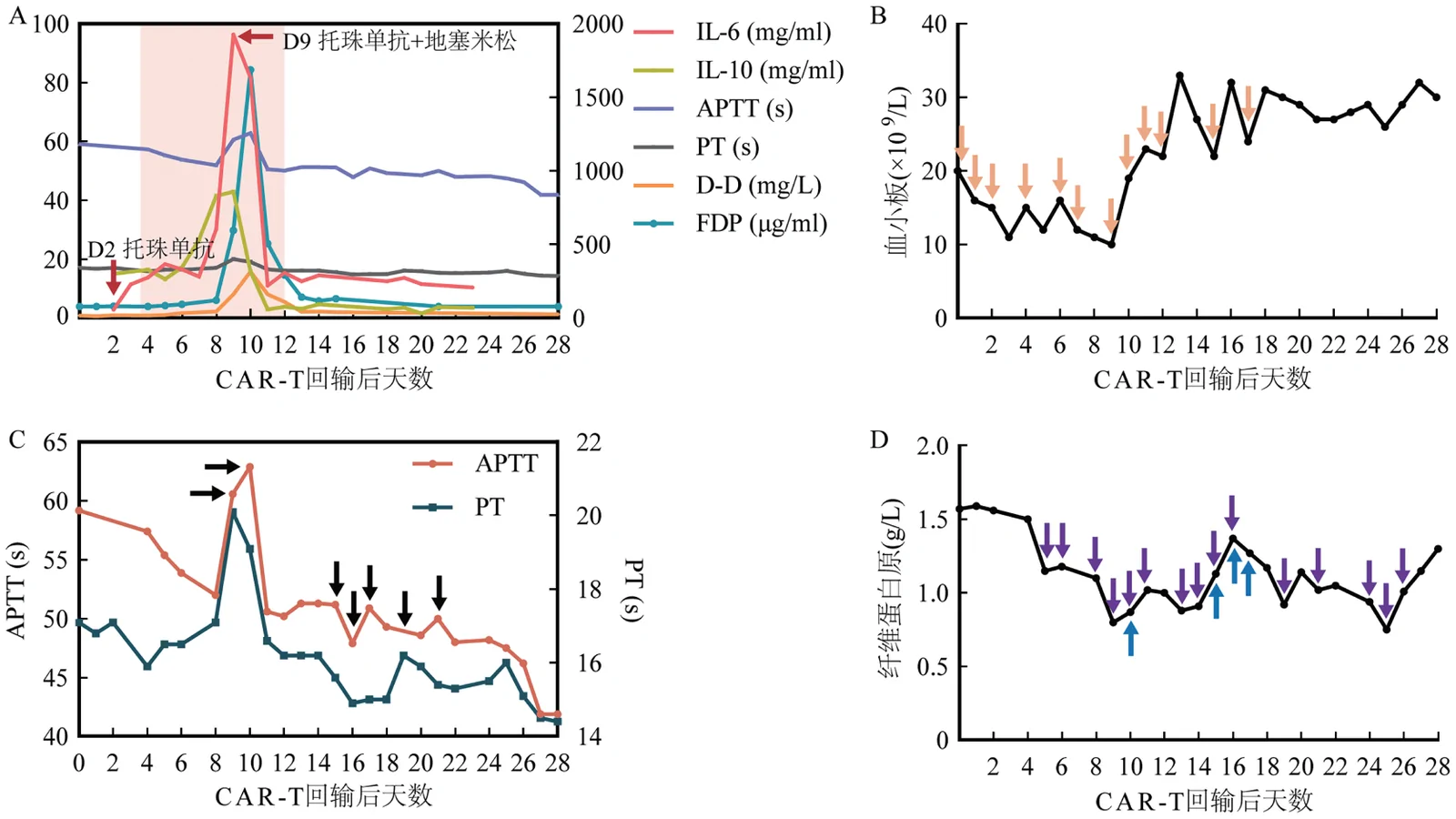
该患者在CAR-T细胞回输后28 d内的炎症和凝血指标及治疗措施 **A** 炎症因子及部分凝血指标水平；**B** PLT水平；**C** APTT和PT水平；**D** 纤维蛋白原水平 **注** A图左Y轴代表IL-10、APTT、PT、D-D、FDP的水平，右Y轴代表IL-6的水平；红色箭头代表在患者发生细胞因子释放综合征时，给予托珠单抗以及地塞米松处理；B图橙色箭头代表在患者发生血小板减少时给予输注血小板治疗；C图黑色箭头代表在患者发生凝血障碍时给予输注血浆治疗；D图紫色和蓝色箭头分别代表在患者纤维蛋白原水平降低时，分别给予补充纤维蛋白原和冷沉淀处理。IL-6：白细胞介素-6；IL-10：白细胞介素-10；APTT：活化部分凝血活酶时间；PT：凝血酶原时间；D-D：D-二聚体；FDP：纤维蛋白降解产物

**图2:**
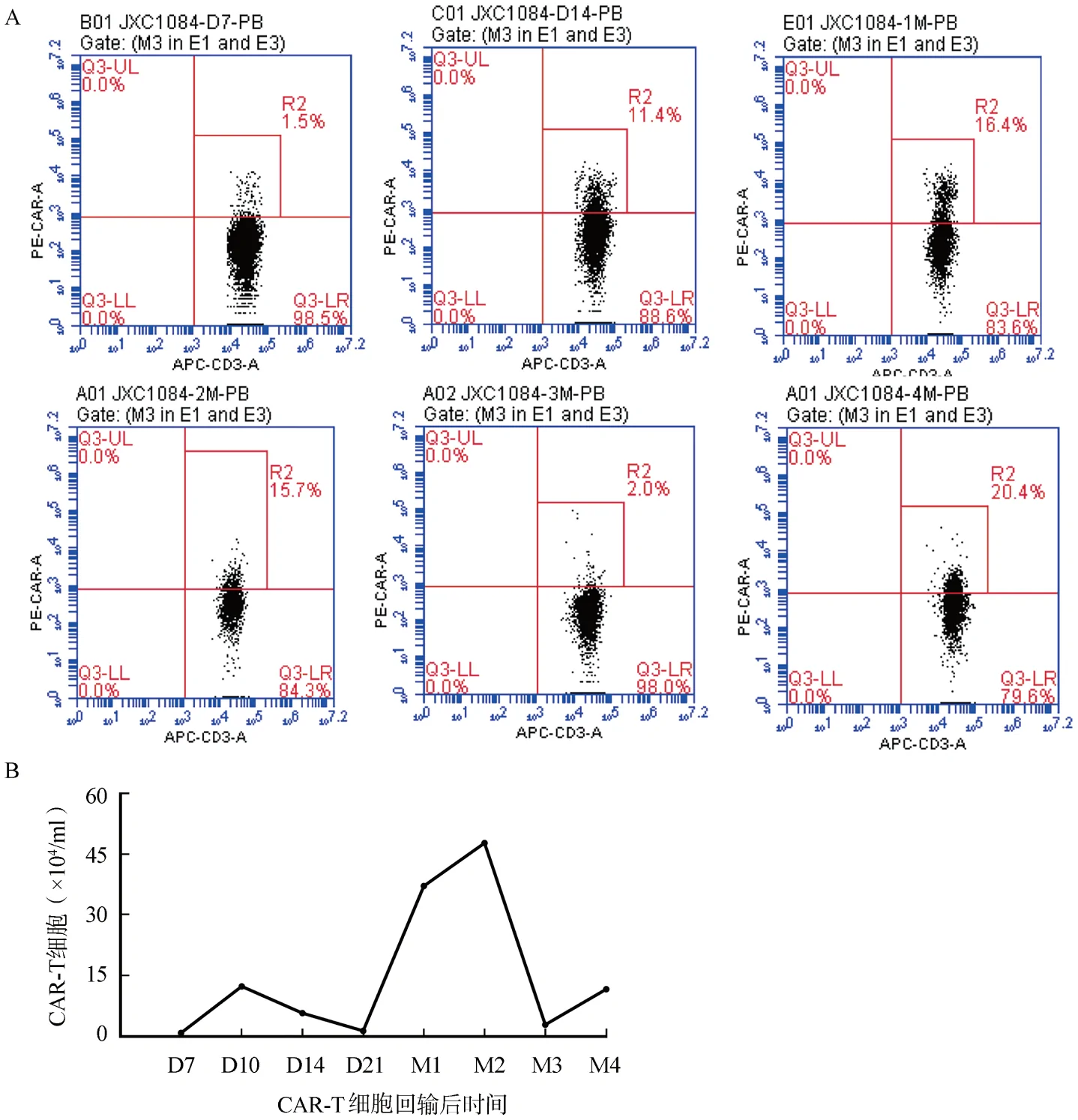
该患者在CAR-T细胞回输后随访期间的体内CAR-T细胞水平 **A** 体内CAR-T细胞比例；**B** 外周血CAR-T细胞绝对计数

## 讨论及文献复习

近年来，CAR-T细胞疗法在肿瘤治疗领域取得了突破性进展[7]。然而在取得显著疗效的同时，CAR-T细胞疗法也伴随着CRS、神经毒性、出凝血功能障碍等并发症，严重时会威胁患者生命健康，增加社会经济负担[4],[8]。本团队于2016年率先报道了一例B-ALL患儿接受CAR-T细胞治疗后发生凝血功能异常的病例[9]。随后的临床研究发现，50％～56.6％的血液肿瘤患者可能发生CAR-T相关出凝血障碍，出血是最常见的临床表现，发生率为9.4％～19.6％，出血部位包括颌面部、软组织、生殖系统、胃肠道、呼吸道以及颅脑等[10]–[12]。血栓性事件也有报道，发生率为2.6％～11％，主要包括深静脉血栓、肺栓塞、血栓性中风、门静脉血栓以及脾静脉血栓等[10],[13]–[14]。值得注意的是，根据中国DIC诊断积分系统和国际血栓止血协会诊断标准，CAR-T治疗后发生出凝血障碍的患者中，有14％～60％进展到DIC，其中6.7％～42.9％的DIC患者死亡[11],[14]–[17]，提示CAR-T相关出凝血障碍是一种危及生命的并发症，应引起临床医师的注意。国内CAR-T治疗及出凝血领域的专家于2022年制订共识，将CAR-T相关出凝血障碍（CAR-T-associated coagulopathy，CARAC）定义为一种在CAR-T细胞回输后短期内发生（绝大多数在28 d内），与细胞因子释放相关，以出血和（或）血栓为特征，伴血小板水平下降和凝血指标异常的临床综合征[4]。凝血指标的异常通常发生在临床表现之前。一项及以上凝血指标异常的发生率为33％～91％，包括纤维蛋白原水平降低（17％～62％）、D-二聚体升高（39％～81％）、纤维蛋白原降解产物升高（45％）、PT延长（4％～46％）、APTT延长（17％～84％）、凝血酶时间延长（7％～79％）[18]。本病例中，患者出现显著的低纤维蛋白原血症、D-二聚体和纤维蛋白降解产物升高、APTT延长，与既往研究一致，提示CARAC是一种消耗性凝血病[11],[15]–[16],[19]–[20]。

CARAC的具体机制尚未完全阐明，目前认为其与CRS密切相关。一项针对复发/难治性急性B淋巴细胞白血病的临床研究表明，凝血指标的异常通常继发于IL-6等细胞因子的显著升高，与CRS的严重程度呈正相关，并在CRS得到控制后逐渐缓解[15]。另一项针对复发/难治性多发性骨髓瘤的临床研究发现，IL-6和干扰素-γ与D-二聚体、APTT、PT的峰值呈正相关，而与纤维蛋白原和血小板的谷值呈负相关[19]。大量细胞因子可激活内皮细胞，进一步促进IL-6、IL-8以及单核细胞趋化蛋白-1等炎症因子分泌，形成细胞因子风暴的恶性循环[21]。内皮屏障的破坏不仅降低了内皮的抗凝活性，还通过释放组织因子触发凝血级联反应，导致纤溶系统过度活化，从而引发凝血-纤溶系统失衡，表现为持续性的低纤维蛋白原血症[15],[20]。高瘤负荷也是CARAC的重要危险因素[11],[16],[19]。高瘤负荷可加剧CRS的严重程度，同时大量肿瘤细胞裂解释放的促凝物质进入血液，可诱发凝血系统的过度激活[22]。此外，CAR-T疗法可能引起肝功能损伤，导致凝血-纤溶相关蛋白的合成减少，进一步加重CARAC[23]。

根据中国专家共识，CARAC的治疗原则包括早期识别、准确评估、去除诱因、分层干预[4]。CARAC的高危因素包括高肿瘤负荷、CAR-T细胞高速扩增及高级别CRS等。对于高危患者，一旦患者在CAR-T细胞回输后开始出现凝血指标异常，应每天使用中国DIC诊断积分系统或国际血栓与止血协会积分系统对其进行DIC评分，以确定DIC的起止时间点，动态监测DIC的严重程度，及时调整治疗方案[6],[24]。对于CARAC患者，应予以控制CRS、替代治疗、抗凝和抗纤溶等治疗[4]。当PLT<20×10^9^/L或PLT<50×10^9^/L但合并活动性出血，应输注血小板悬液[25]。当PT延长≥3 s和/或APTT延长≥10 s，应输注新鲜冰冻血浆10～15 ml/kg，或酌情输注凝血酶原复合物[25]。对于≥3级CRS伴CARAC患者，应每日监测纤维蛋白原水平，酌情补充纤维蛋白原浓缩物或冷沉淀，治疗目标是维持纤维蛋白原水平≥1.5 g/L，直到CRS低于3级[20],[26]。当纤维蛋白原<1.5 g/L，建议纤维蛋白原浓缩物补充剂量为［（1.5−纤维蛋白原实际测量值）/0.017］mg/kg[20]。本例患者在CAR-T回输后第4天开始发生CARAC，其中第9至10天诊断为DIC，从CAR-T回输当天至回输后第21天，累计输注血小板15人份，血浆4200 ml，冷沉淀43 U，纤维蛋白原24 g。

对于需要治疗的CARAC患者，约79％在接受托珠单抗、糖皮质激素和替代治疗后病情好转，但部分DIC患者疗效不佳并死亡[11]。因此，及早预测CARAC的发生显得尤为重要。2022年，Galli等[14]发现校正的内皮激活应激指数评分（modified endothelial activation and stress index，mEASIX，计算公式为C反应蛋白×乳酸脱氢酶/血小板）与异常凝血指标呈正相关，并提出mEASIX ≥6.89可预测CAR-T细胞回输后两周内DIC的发生。然而这个研究的队列规模较小，且mEASIX预测DIC的敏感性较低（53％）。2023年本团队研究发现，预处理前的IL-10联合EASIX可有效预测出血事件的发生，并实现出血危险分层[12]。总体而言，目前尚无高性能的预测模型能够对CARAC发生及危险分层进行早期预警，这也是未来研究的重要方向。
